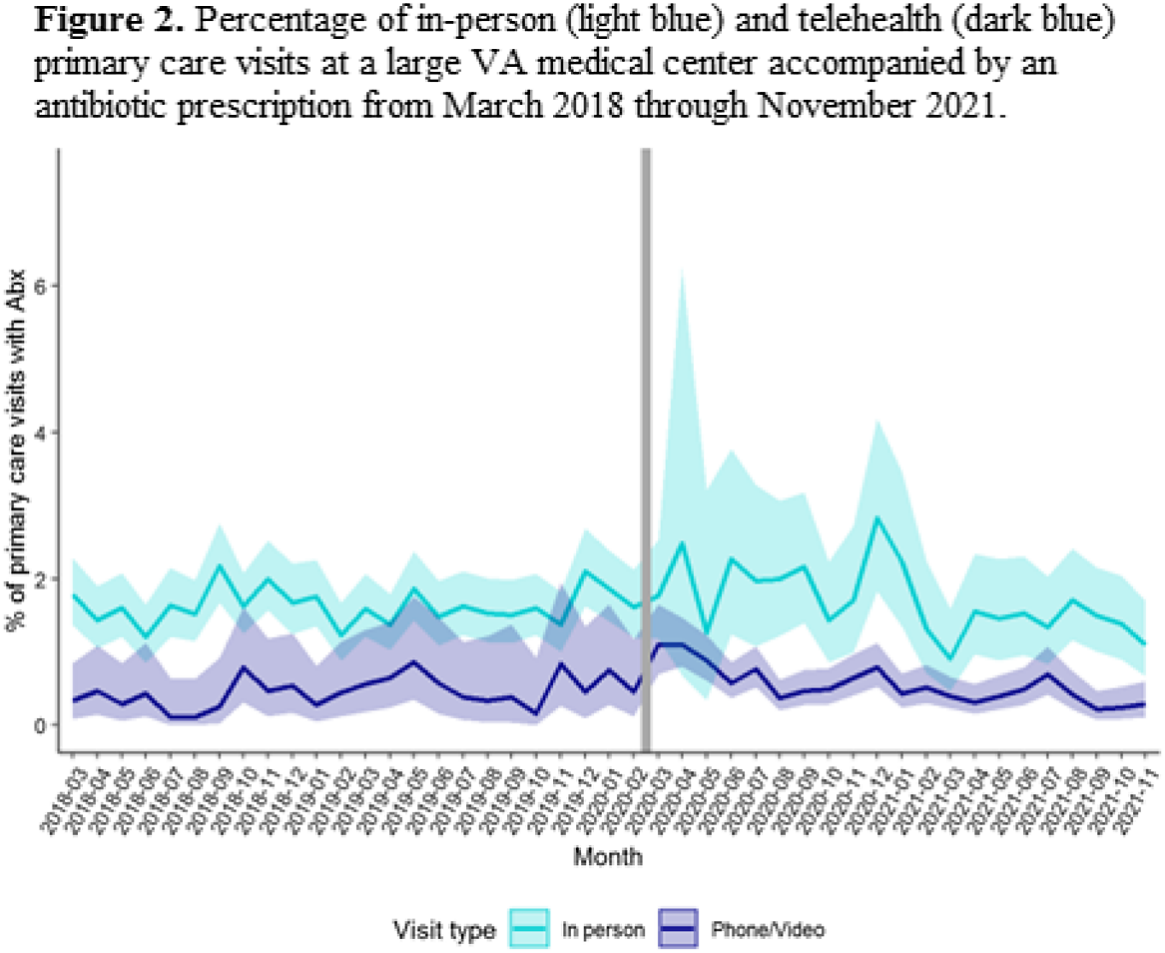# Outpatient antibiotic use for common infectious diagnoses: Patterns in telehealth during the emergence of COVID-19

**DOI:** 10.1017/ash.2022.57

**Published:** 2022-05-16

**Authors:** Nicole Mongilardi, Brigid Wilson, Taissa Bej, Sunah Song, Federico Jump, Federico Perez, Ukwen Akpoji

## Abstract

**Background:** The Veterans’ Affairs (VA) healthcare system has had established telehealth programs for several years. Even so, the COVID-19 pandemic led to an expansion of and changes in these services. Little is known about the influence of the increased use of telehealth due to the COVID-19 pandemic on antibiotic prescriptions in outpatient settings. Here, we report on changes in visit modality and antibiotic prescribing at primary care clinics at a large VA medical center after the emergence of the COVID-19 pandemic. **Methods:** Using VA administrative databases, we identified primary care visits from March 2018 to November 2019 (before the COVID-19 pandemic) and March 2020 to November 2021 (during the COVID-19 pandemic), which permitted us to account for seasonality while analyzing visit modality and antibiotic trends. For primary care visits during the pre–COVID-19 and COVID-19 periods, we have described the type of visit (in-person or telehealth), diagnostic codes for any infection, and antibiotic prescriptions. **Results:** The patient population was primarily men (89%) with a mean age of 62.9 years (SD, ±15.3) at first visit. The most common medical conditions were diabetes mellitus (26%) and chronic lung disease (17%). Comparing visits during the pre–COVID-19 and the COVID-19 periods, the proportions of telehealth visits were 20% (17,708 of 88,565) and 74% (69,891 of 94,937), respectively (Fig. 1). The proportions of visits with an antibiotic prescription were 1.4% (1,212 of 88,565) and 0.8% (798 of 94,396), respectively. When considered by the type of visit, the rates of antibiotics prescribed were consistent during the pre–COVID-19 and COVID-19 periods, with a lower rate for telehealth visits (Fig. 2). In both periods, >50% of antibiotic prescriptions occurred during visits without an associated infectious disease diagnosis. **Conclusions:** Compared to the pre–COVID-19 period, primary care providers at a large VA medical center prescribed fewer antibiotics during the COVID-19 period, and they saw most of their patients via telehealth. These results suggest that some aspects of telehealth may support clinical practices consistent with antibiotic stewardship. The prescription of an antibiotic without an associated diagnostic code also suggests opportunities to improve implementation of antibiotic stewardship principles in primary care settings.

**Funding:** This work was supported by the Merck Investigator Studies Program (grant no. MISP 59266 to F.P. and R.J.) and by funds and facilities provided by the Cleveland Geriatric Research.

**Disclosures:** None

**Figure:**
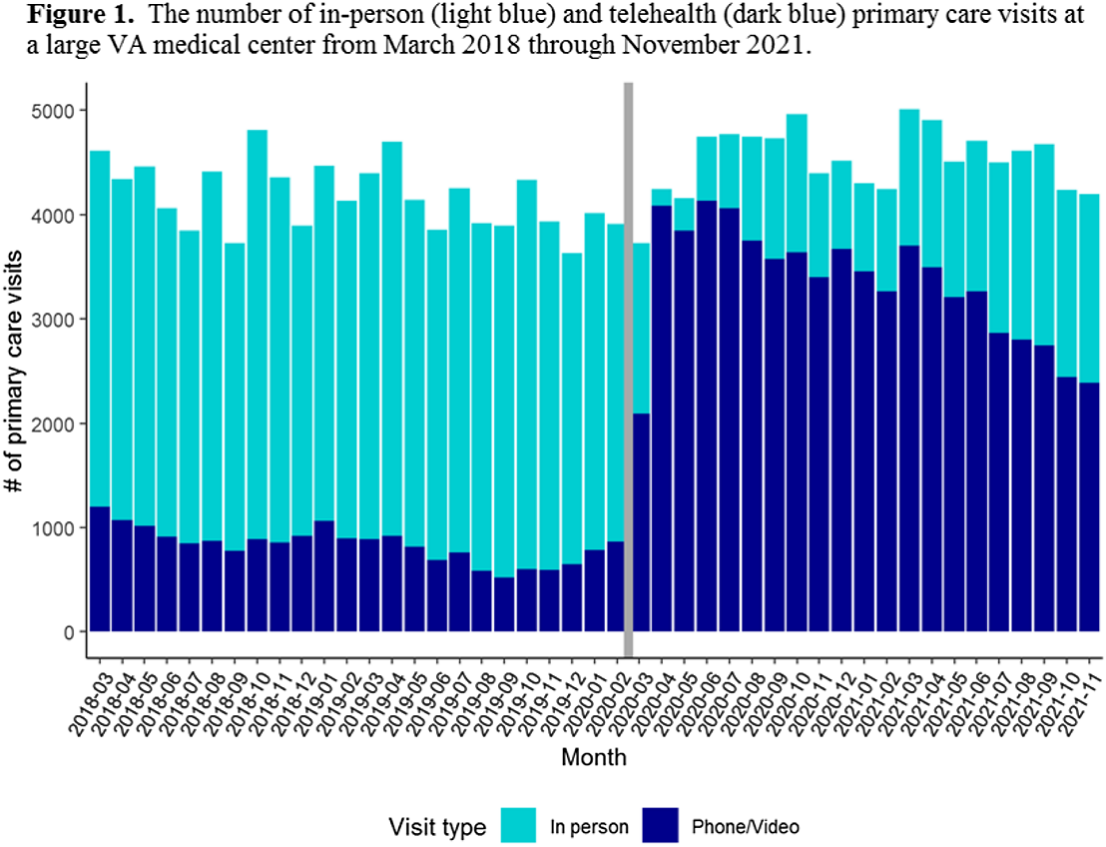

**Figure:**